# Removal of 4-Chlorophenol from Contaminated Water Using Activated Carbon from Dried Date Pits: Equilibrium, Kinetics, and Thermodynamics Analyses

**DOI:** 10.3390/ma9040251

**Published:** 2016-03-30

**Authors:** Hussein Allaboun, Fahmi A. Abu Al-Rub

**Affiliations:** 1 Chemical Engineering Department, Jordan University of Science and Technology, P. O. Box 3030, Irbid 22110, Jordan; abualrub@just.edu.jo; 2 Jordan Atomic Energy Commission, P. O. Box 70, Amman 11934, Jordan

**Keywords:** adsorption, activated carbon, 4-chlorophenol, toxic-pollutants removal, water purification

## Abstract

Five different activated carbons (ACs) have been prepared from dried date pits using air and phosphoric acid as activating agents. The used phosphoric acid:date pit ratio dictated the characteristics of the prepared ACs; the equivalent BET-nitrogen surface area varied from 794 m^2^/g for a ratio of 5:1, to 1707 m^2^/g for a ratio of 2:1, whereas the micropore volume changed in value from 0.24 cm^3^/g for the 5:1 ratio to 0.59 cm^3^/g for the 2:1 ratio. The prepared ACs were tested to remove 4-chlorophenol (4-CP) from aqueous solutions by means of batch adsorption process. The prepared 2:1 AC exhibited the highest uptake with a maximum of 525 mg/g. Equilibrium pH studies showed that 4-CP removal was pH dependent; the maximum uptake occurred at an equilibrium pH value of 5.5. Dynamic studies showed that 4-CP uptake on 2:1 AC is rapid, with 80% of the maximum uptake achieved during the first 40 min. Both surface adsorption and intraparticle diffusion were identified to be effective adsorption mechanisms. Kinetic studies indicated a pseudo second-order reaction. Results of equilibrium adsorption experiments showed that the adsorption of the 4-CP on 2:1 AC is best described by the Langmuir model. The thermodynamics parameters of the adsorption (Δ*G*^0^, Δ*H*^0^, and Δ*S*^0^) were determined by studying the adsorption equilibrium at different temperatures. The values of these parameters indicated the spontaneous and endothermic nature of the adsorption phenomenon of 4-CP on the prepared ACs.

## 1. Introduction

Chlorophenols and other phenol-based compounds have come to represent some of the most dangerous and persistent organic pollutants because of their various industrial applications [1]. Many of these compounds are byproducts of industrial processes including pharmaceutical, pesticide, paint and solvent production, and wood, paper, and pulp processing. Additionally, as a result of the widespread agricultural use of these compounds as herbicides, insecticides, and fungicides, phenolic pollutants have been detected in many sources of wastewater and drinking water [2,3]. Chlorophenols are easily absorbed after ingestion [4], and while they do not tend to bioaccumulate, the toxicity of these chemicals is well known [5]. Many phenolic compounds have been identified as possible carcinogens; long-term exposure has been found to cause liver, kidney, and neurological defects [5].

For obvious reasons, the removal of chlorophenols, and the precursor phenols, from polluted areas and water sources is of utmost importance. Many methods for their removal have been investigated, including biological treatment with microorganisms [6,7,8] and enzymes [9,10,11]; and via adsorption using organobentonites [12], zeolite [13], fly ash [14], and many sources of activated carbons [15,16]. Additionally, various catalytic methods have been employed, such as those involving photocatalytic degradation [17,18] and electrochemical oxidation [19].

Activated carbon has proven to be a very effective adsorbent for many types of inorganic and organic compounds because of its high surface area and unique chemical properties, including the polarity and nature of surface functional groups. The surface chemistry of these carbons and the chemical characteristics of adsorbate, such as polarity, ionic nature, functional groups, and solubility, determine the nature of the adsorption mechanism, as well as the extent and strength of adsorption. Moreover, the porous structure of activated carbon consists of a network of interconnected macropores, mesopores, and micropores that provide a good capacity for the adsorption of organic molecules. Thus, it is expected that various mechanisms and forces, such as ion exchange, covalent bonding, van der Waals forces, H-binding, dipole-dipole interactions, and cation- and water-bridging, can be responsible for adsorption of organic compounds in activated carbon [20,21,22,23,24]. Despite its many advantages, though, the production and regeneration of activated carbon is very expensive, with higher grades commanding even higher costs. In an attempt to find alternative, more economical, techniques many researchers have explored the use of other sorbents [25,26,27], with varying degrees of success [24]. Alternatively, activated carbons prepared from inexpensive and plentiful raw materials could prove to be an affordable, yet still very effective, solution [20,21,22,23]. Activated carbon from agricultural sources, usually wastes, can be prepared either by physical activation, which involves primary carbonization (below 700 °C) followed by controlled gasification under the action of oxidizing gases at high temperature (up to 1100 °C), or chemical activation where the precursor is mixed with a chemical that restricts the formation of tars (e.g., nitric acid, phosphoric acid, hydrogen peroxide, *etc.*). After kneading, the precursor is carbonized and washed to produce the final AC. The chemical incorporated into the interior of the precursor particles reacts with the thermal decomposition products reducing the evolution of volatiles and inhibiting the shrinkage of the particles. This increases the conversion of the precursor to carbon and produces carbons with large internal porosity. After the heat treatment, the chemical is removed by copious washing [20]. In the physical activation, the active carbon is prepared by activating the carbonized intermediate products with gaseous agents. The carbonized intermediate product is prepared by carbonization of the carbonaceous material at higher temperatures. Steam, carbon dioxide, and oxygen are the most common activating agents used. During the activation of the carbonized intermediate product first the disorganized carbon is removed, and by this the surface of the elementary crystallites becomes exposed to the action of the activating agent. The burning out of the crystallites must proceed at different rates on different parts of the surface exposed to reaction; otherwise new pores could not be formed [28]. A possible explanation of the mechanism is that the velocity with which the crystallites burn is larger in the direction parallel with the plane of the carbon layers than in the direction perpendicular to this plane [29]. The crystallites orientated perpendicularly to the surface exposed to the action of the activation agents can, therefore, be more easily attacked and burn out more quickly [28].

Moreno-Castilla [30] investigated in a comprehensive study the adsorption of organic molecules from dilute aqueous solutions on carbon materials. He concluded that the adsorption process is a complex interplay between non-electrostatic and electrostatic interactions. He showed that “Non-electrostatic interactions are essentially due to dispersion and hydrophobic interactions, whereas the electrostatic or coulombic interactions appear with electrolytes when they are ionized at the experimental conditions used. Both interactions depend on the characteristics of the adsorbent and the adsorptive and the solution chemistry.” A multitude of studies has investigated the surface properties and porous structure characteristics of activated carbons derived from natural residues using phosphoric acid [31,32,33,34,35]. Preparation methods comprised chemical and/or physical activation mechanisms. Tailored textural properties and surficial characteristics were possible by applying the appropriate conditions [32].

Palm dates grow abundantly in tropical regions and the gulf countries of the Middle East; after processing the fruits, the residual stones, which constitute approximately 10% of the weight of the dried fruit [36], represent a significant agricultural byproduct in these countries. As an economical solution to the problem associated with this waste, activated carbon with high surface areas can be prepared from date pits [37]. Studies have shown that date pit-derived activated carbon is effective as an adsorbent for phenol and heavy metal contaminants [38,39]; here, we investigate its use in the sorption of 4-chlorophenol (4-CP) from aqueous solutions.

In our previous study, we used the activated carbon prepared from date pits to remove phenol from aqueous solution. The study involved dynamics and equilibrium analysis only. For this study, raw date pits were carbonized and activated by air and phosphoric acid; the resulting activated carbon was evaluated for its 4-CP sorbent capacity. The effects of solution pH on adsorbent capacity were investigated and the Langmuir and Freundlich models were utilized to describe the adsorption behavior. Parameters for each of these models were determined by non-linear error estimation using the derivative of Marquarrdt’s Percent Standard Deviation (MPSD). The pseudo second-order kinetics and Elovich equation, in addition to the Weber and Morris equation, and diffusion models were used to analyze the dynamics of sorption of the 4-CP on the prepared AC. Thermodynamics analysis of the 4-CP adsorption was carried out using both the Arrhenius and van’t Hoff equations.

## 2. Materials and Methods

### 2.1. Materials and Instrumentation

Five different activated carbons were prepared from date pits according to a method discussed in our previous work [20]. The approximate composition of the date pits used is shown in Table 1. The date pits were milled using a coffee mill. The resulting powder was sieved through a standard 150-mesh sieve. The small size of the powdered date pits enabled the highest possible exposure of these particles to oxygen and phosphoric acid and, therefore, increased functional group development. The powdered date pits (C) were mixed with 85% phosphoric acid (A) in different weight ratios (A:C) of 1:2, 1:1, 2:1, 3:1, and 5:1, and diluted five times with deionized water in a 1000 mL beaker. The beaker was placed on the hot plate/stirrer and gently boiled for a few hours until the mixture turned to a black paste. The temperature of the paste at this stage was 160 °C. In the same beaker, the heating continued while the material was stirred with a glass tube that introduced air at a flowrate of 3.0 L/min. This stage of carbonization and activation continued for 15 min and the temperature increased to approximately 215 °C. The black paste (activated carbon) produced was poured into a test tube that was placed vertically in a tubular furnace. It was activated for 30 min with an airflow rate of 2.5 L/min at a temperature ranging from 300 to 500 °C. After activation, the tube was removed from the furnace and cooled for 10 min with the flowing air. The synthesized AC was washed by boiling in deionized water, filtered, and then rinsed with deionized water. This procedure was repeated twice, then the sample was placed in a soxhlet extractor and rinsed with deionized water for 48 h. The rinsed carbon was transferred to a beaker and slurried with deionized water. The slurry was boiled and filtered. The wash water was tested with lead nitrate solution to ensure that all phosphate had been removed. After cooling, the pH of the wash water was measured to assure neutrality. The carbon was dried in a vacuum oven at 100 °C for 12 h, cooled in a dessicator, weighed, ground, transferred to a container, and stored in the dessicator [20].

**Figure 1:**
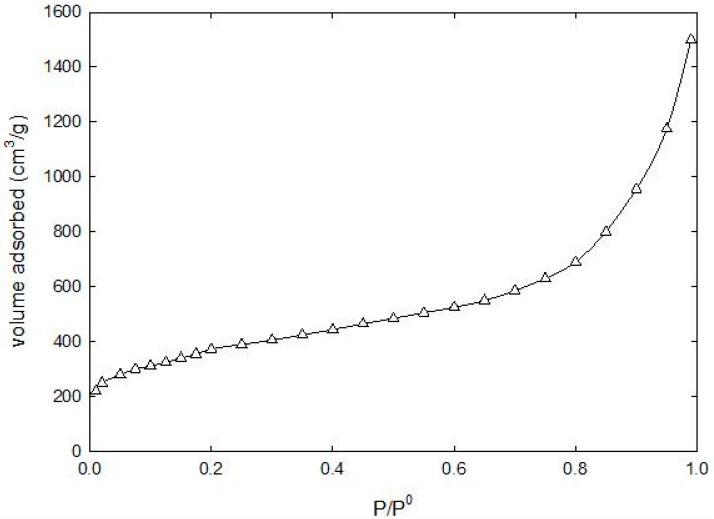
N_2_ Adsorption isotherm on 2:1 AC at 77 K.

Samples of the prepared ACs were tested using a Micrometrics ASAP 2010 (Company, City, Country) (Micrometrics Instrument corporation, Atlanta, GE, USA) gas adsorption surface area and porosimetry analyzer. In this test, nitrogen adsorption isotherms at 77 K were obtained, as shown in Figure 1. The manufacturer’s software provided was used to determine the total pore volume (Vpore), the equivalent BET-nitrogen surface area of the adsorbent using the BET equation [2], and total volume of pores was calculated at a relative pressure (P/P0) of 0.99. The functional groups on the prepared ACs were determined by the Boehm’s titration method, which was discussed in detail in our previous work [20]. Table 2 lists the different functional groups and relative concentration (M_eq_H^+^/g) of functional groups available on the 2:1 AC.

The point of zero charge was determined using the acid–base titration method developed by Newcombe *et al.* [40]. Several 50-mL portions of 0.01 M NaCl solution were prepared in flasks and the required pH for each portion was adjusted by the addition of the appropriate amount of 0.01 M solutions of either NaOH or HCl. When the required pH was achieved, 0.15 g of the prepared activated carbon sample was added to each flask and shaken for 24 h, and the final pH was recorded. Control tests were conducted without the use of activated carbon samples to eliminate the influence of CO_2_ on pH. The point of zero charge (pH_pzc_) was defined as the point where the pH final *vs.* pH initial curve crosses the line pH_initial_ = pH_final_.

### 2.2. Adsorption Experiments

Batch adsorption experiments were conducted by adding the appropriate amount of the prepared ACs into 100 mL reagent bottles containing 50 mL portions of 4-CP solution. Different initial concentrations of the 4-CP (10–400 ppm) were used in the equilibrium studies. The experimental procedures for measuring adsorption kinetics, equilibrium, and thermodynamics are described and discussed in detail in our previous studies [20]. The concentration of the 4-CP was determined using a UV-VIS spectrophotometer at 279 nm.

The adsorption capacity (*i.e.*, uptake) was calculated using Equation (1):

(1) $$q_{e}=\frac{\left(C_{0}-C_{e}\right)V}{w}$$

where *q_e_* is the equilibrium uptake (mg/g); *C_o_* is the initial 4-CP concentration (mg/L); *C_e_* is the equilibrium 4-CP concentration (mg/L); *V* is the volume of the solution (L); and *w* is the mass of the adsorbent (g).

## 3. Results and Discussion

### 3.1. Characterization of the Prepared ACs

**Figure 2:**
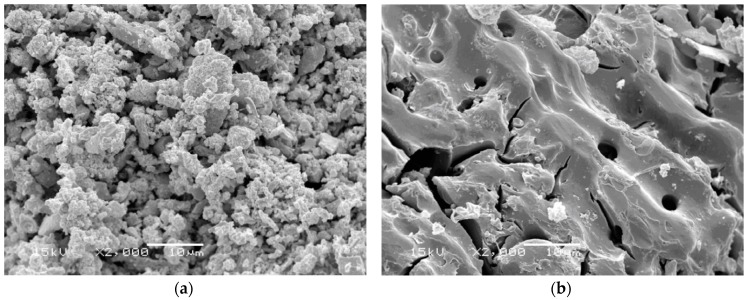
SEM images of 2:1 AC (**a**) and 5:1 AC (**b**) preparations.

The equivalent BET-nitrogen surface area, molecular volume, total pore volume, micropore volume, and pore diameter of the five prepared ACs were determined and listed in Table 3. SEM representative images of these ACs are shown in Figure 2. It is evident from the results of the SEM images and the BET data that the surface area of the prepared ACs increases as the ratio of phosphoric acid to precursor decreases, until it reaches the ratio of 2:1 (A:C) where it, then, starts to decrease. This trend can be attributed to the enlargement of the micropores until they are damaged by the effect of excessive amounts of the acid. Table 3 shows that acid activation enhanced the porosity of all ACs which resulted in a pronounced increase in the total pore volume and specific surface area for all activated carbons. These results can be explained on the basis of different activation mechanisms, namely the pyrolysis of the raw materials enhanced the cross-linkage and, hence, created highly porous materials, while activation with phosphoric acid led to the elimination of water and, therefore, destruction of the cellulosic structure [20,41].

The increase of the extent of activation from date pits to activated carbon was associated with the increase in total pore volume and specific surface area and a decrease in the mean pore radius; suggesting the significant contribution of micropores to the total pore volume. However, in the case of activation for 2:1–5:1 ACs, different results were observed where the surface area and total pore value was drastically decreased and the result for pore diameter was ambiguous. This may be attributed to the fact that excess acid could form a protective solid layer that reduces the activation of the raw material. Due to the mixed micro/mesoporosity, the active carbons are not freely accessible to the probe molecules from aqueous solution [20].

All adsorption experiments on 4-CP will be conducted using the 2:1 AC as it has the highest equivalent BET-nitrogen surface area.

### 3.2. Effect of pH on Adsorption of Phenol on 2:1 AC

Most of the studies on the adsorption of aromatic compounds by activated carbons could not completely explain the mechanism by which these compounds are adsorbed. This is attributed to the fact that many variables are involved in the adsorption process, such as the solubility of the aromatic compounds, the ionization state of the functional groups on the adsorbent, electrostatic interactions, dispersive and chemical interactions, and intrinsic properties of both the solute and adsorbent.

**Figure 3:**
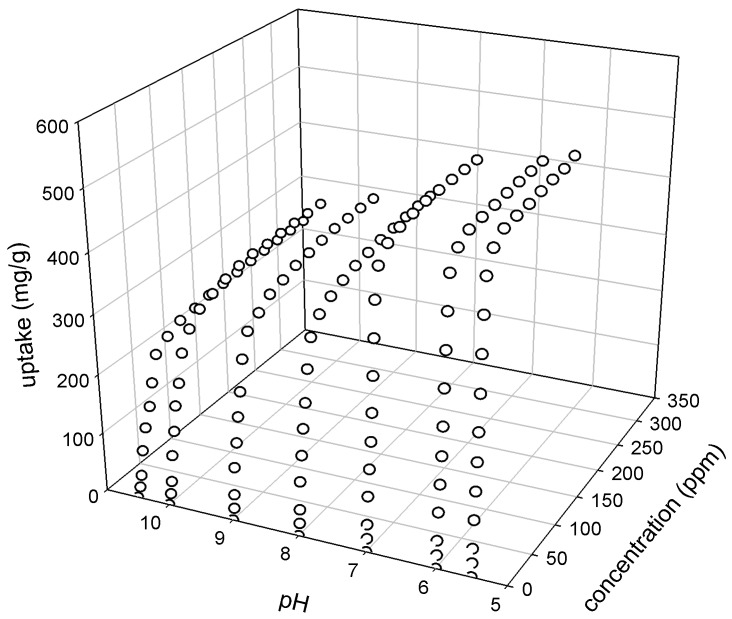
Effect of pH on adsorption of 4-CP on the prepared 2:1 AC (Time = 6 h, mass of AC = 0.20 g).

Adsorption of 4-CP on 2:1 AC was studied over the range of pH 5.5–10.5, and the results are shown in Figure 3. The point of zero charge (pH_PZC_) of the 2:1 AC was found to be 7.2 following the procedure outlined earlier in the materials and methods section. Figure 3 shows that the uptake of 4-CP on the AC reached a maximum at pH 5.5 and it decreased with the increase in solution pH. The decrease in 4-CP uptake was more pronounced at pH > 7.5. This is due to the dependency of the 4-CP ionization on solution pH and the point of zero charge for mesoporous carbon, as well as the electrostatic and dispersive interactions between the 4-CP and the activated carbon.

According to Moreno-Castilla [30], the solution pH also controls the issociation or ionization of the electrolyte through its pKa. Thus, acidic electrolytes will be dissociated at pH > pKa. “Therefore, the solution pH controls the adsorptive-adsorbent and adsorptive-adsorptive electrostatic interactions, which can have a profound effect on the adsorption process. Thus, the adsorption of substituted phenols on activated carbon depends on the solution pH.” For 4-CP, the relative amount of 4-CP, which has a pKa of 9.43, as dissociated chlorophenolate species increases with pH. At pH 5.5, the 4-CP uptake was maximal because the 4-CP was undissociated and the dispersion interactions predominated. However, at basic pH, and particularly at pH > pHPZC, the uptake of the 4-CP was lower because of electrostatic repulsions between the negative surface charge and the chlorophenolate anions and between chlorophenolate-chlorophenolate anions in solution.

### 3.3. Dynamics of 4-CP Adsorption on 2:1 AC

**Figure 4:**
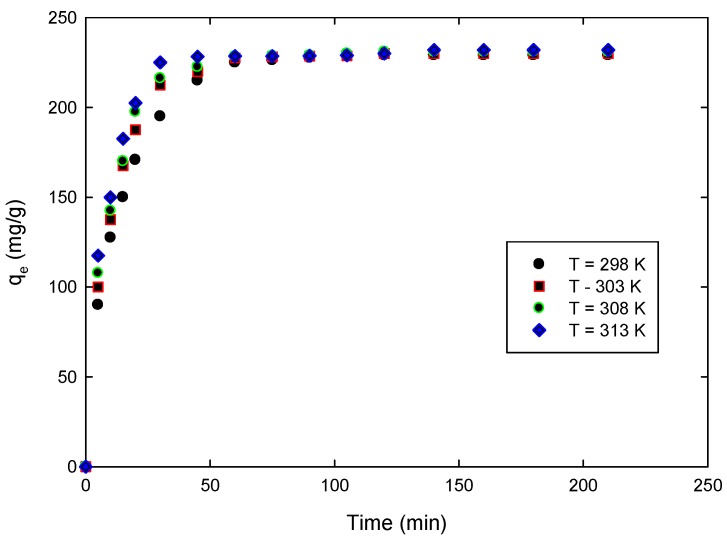
Effect of contact time on adsorption of 4-CP on the prepared 2:1 AC (pH = 5.5, initial concentration of 4-CP = 100 ppm, mass of AC = 0.20 g).

Dynamics of the adsorption of 4-CP on the prepared ACs was investigated with 100 ppm 4-CP solution on 2:1 AC at the optimum pH; *i.e.*, 5.5, and at different temperatures. The results of the change of 4-CP uptake with time are presented in Figure 4.

It is evident from this figure that the uptake of 4-CP on 2:1 AC is rapid; more than 85% of the maximum uptake occurs during the first 40 min. After 40 min the adsorption process plateaus and becomes slower, near equilibrium. In between these two stages of uptake, the rate of adsorption is found to be nearly constant. This can be explained based on the fact that during the first 40 min, a large number of vacant surface sites are available for adsorption; thus, the uptake increases rapidly. After 40 min, the remaining vacant surface sites are sterically and electrostatically hindered due to the repulsive forces between the solute molecules on the solid and bulk phases; thus, the uptake advances more slowly.

The adsorption of 4-CP on 2:1 AC can be analyzed by the pseudo second-order kinetics given by [42]:

(2) $$\frac{dq_{t}}{dt}=k_{2}{\left(q_{e}-q_{t}\right)}^{2}$$

where *q_e_* (mg/g) is the uptake at equilibrium; *q_t_* (mg/g) is the uptake at time *t* (min); *k_2_* (g/mg·min) is the equilibrium rate constant of pseudo second-order sorption kinetics. Equation (2) can be solved with the boundary condition: $q_{t}{|}_{t=0}=0$, to give Equation (3):

(3) $$\frac{t}{q_{t}}=\frac{t}{q_{e}}+\frac{1}{q_{e}^{2}k_{2}}$$

The adjusted *q_e_* and *k*_2_, can be determined by either fitting the equilibrium data to linear-equivalent forms or using nonlinear regression techniques. In this study, the Composite Fractional Error Function (CFEF) [43], defined as seen in Equation (4), was used:

(4) $$\text{CFEF}=\text{min}{\sum_{i=1}^{P}\left[{\frac{\left(q_{e,\exp-}q_{e,cal}\right)}{q_{e,\exp}}}^{2}\right]}_{i}$$

where $q_{e,exp}$ and $q_{e,cal}$ are the experimentally-determined and calculated values of *q_e_*, respectively. Using CFEF, the parameters of the pseudo second-order rate *k*_2_ and *q_e_*, at different temperatures are listed in Table 4. The low values of CFEF indicate that this model appropriately describes the kinetics of adsorption for the prepared ACs.

Another common model used to describe activated chemical adsorption is the Elovich equation [39]:

(5) $$\frac{dq_{t}}{dt}=a\exp(-bq_{t})$$

where *a* and *b* are the constants determined during an experiment. The constant, *a*, is regarded as the initial rate because ($dq_{t}/dt$) approaches *a* when *q_t_* approaches 0 [44]. Equation (5) can be solved with the boundary condition: $q_{t}{|}_{t=0}=0$, to give:

(6) $$q_{t}=\left(\frac{1}{b}\right)\ln\left(a\cdot b\right)+\left(\frac{1}{b}\right)\ln\left(t\right)$$

The intercept of Equation (6) indicates the amount of adsorption in a short time, as t→0, and the slope indicates the capability of mass transfer and solute adsorption in the interior pores of particles [44].

Using Equation (4), the parameters of the Elovich equation, *a* and *b*, were found and listed in Table 4. The low values of CEFF indicate that this model appropriately describes the kinetics of adsorption for the prepared ACs.

**Figure 5:**
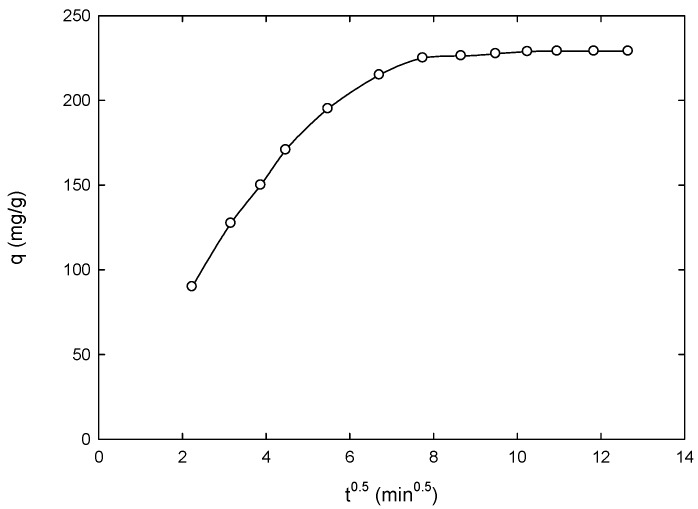
Application of Weber and Morris equation on experimental data for the adsorption of 4-CP on the prepared 2:1 AC (*T* = 298 K, pH = 5.5, concentration of 4-CP = 100 ppm, mass AC = 0.20 g).

To investigate the diffusion mechanism of the 4-CP adsorption onto the carbon, the intraparticle diffusion model described by Equation (7), proposed by Weber and Morris [45], was utilized:

(7) $$q_{t}=k_{id}t^{0.5}+C$$

where, *k_id_* (mg/g·min^1/2^) is the intraparticle diffusion rate constant and *C* (mg/g) is a constant that is related to the thickness of the boundary layer, *i.e.*, the larger the value of *C* the greater is the boundary layer effect. Figure 5 shows the application of Equation (7) to the experimental data for the adsorption of 4-CP on 2:1 AC. Figure 5 indicates multi-linearity rather than linear relation. The multi-linearity of the intraparticle diffusion plots may indicate two or more steps occurring. The first step is characterized by a steep change in the uptake, which may indicate an instantaneous adsorption stage resulting from external surface adsorption, and then followed by sections representing intraparticle diffusion into the branched porous network of the adsorbent particle [46,47].

### 3.4. Adsorption Isotherms

**Figure 6:**
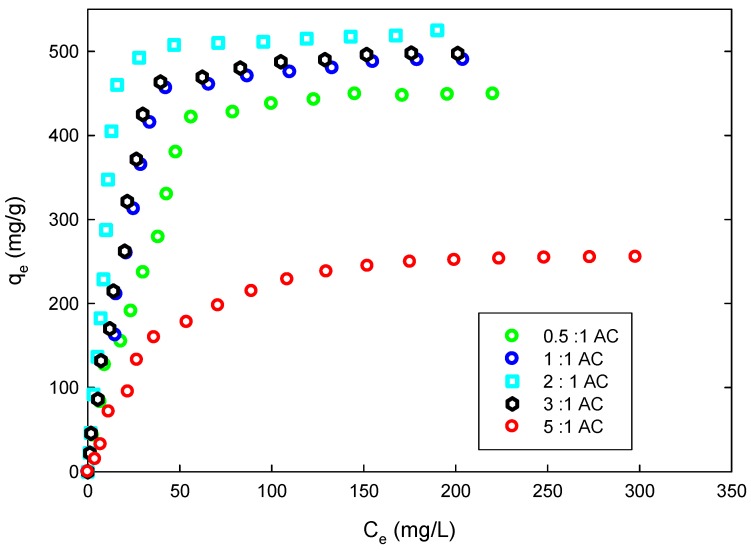
Equilibrium isotherm of adsorption of 4-CP on the prepared ACs (*T* = 298 K, pH = 5.5, mass of AC = 0.20 g).

Adsorption isotherms describe the relationship between the amount adsorbed by a unit mass of solid sorbent and the concentration of dissolved adsorbate in the liquid at equilibrium. Figure 6 indicates that the maximum adsorption was achieved with the use of 2:1 AC. This is expected since the 2:1 AC has the highest surface area.

The Langmuir and Freundlich models are the most common adsorption isotherm models that are used to describe the adsorption equilibrium data. The Langmuir isotherm assumes that: (1) sorption occurs uniformly on the active sites of the sorbent, and (2) once a sorbate occupies a site, no further sorption can take place at this site. Based on these assumptions, the Langmuir isotherm model in its simple form is given by Equation (8):

(8) $$q_{e}=\frac{q_{mon}K_{L}C_{e}}{1+K_{L}C_{e}}$$

where the adjustable parameters *q_mon_* and *K_L_* are the Langmuir constants, which are related to the amount of adsorption corresponding to monolayer coverage, or adsorption capacity, and the energy of adsorption, respectively.

While the Langmuir isotherm model assumes uniform adsorption energies and homogeneous monolayer, the Freundlich isotherm model is given by Equation (9):

(9) $$q_{e}=KC_{e}^{1/n}$$

which assumes that sorption occurs on heterogeneous surfaces. However, it does not provide any information on the monolayer adsorption capacity. The adjustable parameters *K* and *n* are the Freundlich constants and are indicators of adsorption capacity and adsorption intensity, respectively.

**Figure 7:**
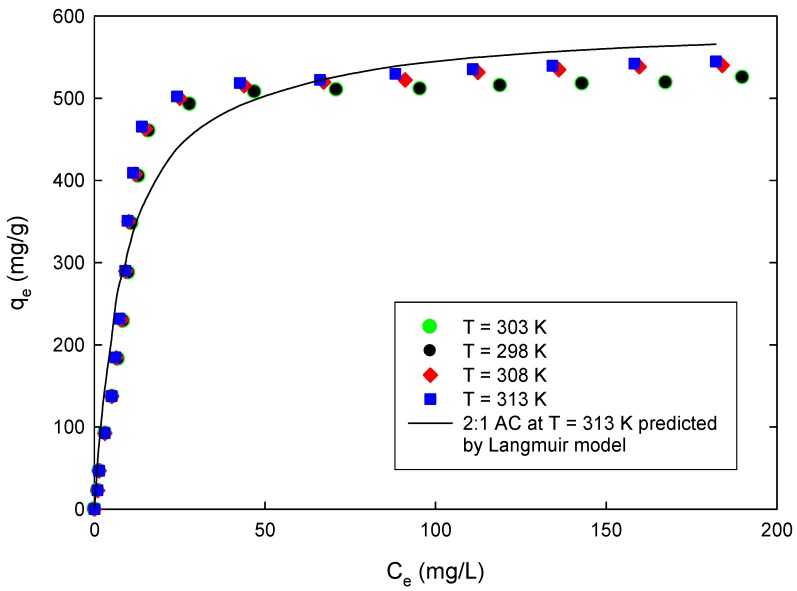
Equilibrium isotherm of adsorption of 4-CP on 2:1 AC (pH = 5.5, mass of AC = 0.20 g).

The adjustable parameters of both the Langmuir and Freundlich models were determined for the adsorption of the 4-CP on the 2:1 AC at different temperatures, as shown in Figure 7, by minimizing the objective function of the derivative of Marquarrdt's Percent Standard Deviation (MPSD) [43] defined by Equation (10):

(10) $$\text{MPSD}=\sum_{i=1}^{n}{\left(\frac{q_{e,exp}-q_{e,cal}}{q_{e,exp}}\right)}_{i}^{2}$$

The parameters, along with the values of MPSD, are listed in Table 5. The values of MPSD indicate that while both the Langmuir and Freundlich models could describe the adsorption of 4-CP on the prepared ACs, the Langmuir gave better fitting to the experimental results.

Table 6 shows a comparison of the Langmuir monolayer adsorption capacity, *q_mon_*, of 4-CP on different adorbents. As it can be noticed, the 2:1 AC is considered among the highest Langmuir monolayer adsorption capacities.

### 3.5. Effect of Temperature and Thermodynamics Analysis

Thermodynamics analysis in sorption studies usually involves studying sorption equilibrium and kinetics at different temperatures. The temperature dependency of the 4-CP adsorption onto the prepared ACs was investigated by studying the effect of temperature on the adsorption equilibrium and kinetics of the adsorption process, as shown in Figure 4 and Figure 7, respectively. The parameters that are important to analyze the thermodynamics of the adsorption process are the standard enthalpy ∆*H*^0^, standard entropy ∆*S*^0^, and standard free energy ∆*G*^0^ due to the transfer of a unit mole of solute from solution onto the solid–liquid interface, as well as the activation energy of adsorption, *E_a_* [52].

The van’t Hoff equation:

(11) $$\mathrm{In}\;K=\frac{-∆H^{0}}{RT}+\frac{∆S^{0}}{R}$$

can be used to determine the value of ∆*H*^0^ and ∆*S*^0^, while ∆*G*^0^ can be calculated using the equation:

(12) $$∆G^{0}=-RT\;\mathrm{In}\;K$$

where K is the distribution coefficient, which can be calculated using the Equation (13):

(13) K=1KL

The activation energy of the adsorption can be determined using the linearization form of Arrhenius equation, given by:

(14) $$\mathrm{In}\;K_{2}=\mathrm{In}\;k_{0}-\frac{E_{a}}{RT}$$

where *K*_2_ is the equilibrium rate constant of pseudo second-order sorption kinetics, *k*_0_ is the Arrhenius factor, and *E_a_* is the Arrhenius activation energy of adsorption. Table 7 lists the calculated thermodynamics properties.

The negative values of ∆*G*^0^ confirms the feasibility of the adsorption of 4-CP on the prepared ACs and the spontaneous nature of the adsorption process. Positive value of ∆*S*^0^ reflects the affinity of the prepared ACs for the 4-CP and the increasing randomness at the solid–solution interface with some structural changes in the adsorbates and adsorbents during the adsorption process. Positive values of ∆*H*^0^ indicate that the adsorption of 4-CP on the prepared AC is endothermic, which agrees with the experimental data where adsorption increases with increasing temperature as shown in Figure 7. The increase of adsorption with temperature can be attributed to the increase of the rate of diffusion of the 4-CP across the boundary layer and in the internal pores of the prepared ACs.

## 4. Conclusions

The activated carbons produced from date pits have proven to be efficient adsorbent for the investigated toxin pollutant, namely 4-Chlorophenol. The adsorption process on the prepared activated carbons was found to be pH dependent; it decreased by increasing pH. Dynamics studies on the prepared activated carbons revealed that Elovich equation appropriately could describe the kinetics of adsorption for the prepared ACs and the process followed pseudo second-order kinetics with the involvement of film, pore, and surface diffusions, as well as adsorption on the pore surface. Equilibrium studies indicated that Langmuir isotherm model best described the adsorption of 4-CP on the prepared activated carbon. The determined thermodynamic properties, indicated the spontaneous and endothermic nature of the adsorption process of 4-CP on the prepared ACs.

## Figures and Tables

**Table 1:** Approximate composition of the date pits used.

Test	mg/kg
Moisture	6.10
Ash	1.17
Crude fiber	9.18
Protein	5.27
Ether extract (crude fat)	1.73
Ca	507
Mg	641
Fe	135
Cu	5.44
Zn	40.7
Al	6.88

**Table 2:** Concentration of surface functional groups on 2:1 AC.

Group	(M_eq_H^+^/g)
Lactones & Phenol	0.013
Lactones	0.005
Carboxyl group	0.005
Acidic	0.023

**Table 3:** Textural characterization of the prepared ACs.

Sample ID	Equivalent BET-Nitrogen Surface Area (m^2^/g)	VM (cm^3^/g STP)	Single Point Adsorption		
			Total Pore Volume (cm^3^/g)	Micropore Volume (cm^3^/g)	Pore Diameter (4 v/A)
1:2 AC	1079	248	0.500	0.46	23.54
1:1 AC	1193	274	0.60	0.48	20.87
2:1 AC	1707	392	1.1	0.59	26.7
3:1 AC	1602	368	1.5	0.47	36.91
5:1 AC	793.	183	0.7	0.24	35.44

**Table 4:** Kinetics parameters for the sorption of 4-CP on the prepared 2:1 AC.

Model	Parameter	Temperature (K)			
		298	303	308	313
pseudo second-order	*q_e_* (mg/g)	243.9	243.9	238.1	238.1
	*k*_2_ (g/mg·min)	0.00064	0.00083	0.00101	0.00101
	CFEF (mg/g)	4.5	5.5	5.1	3.9
Elovich	*a* (mg/g·min)	61.3	76.2	77.9	83.1
	*b* (g/mg)	0.01886	0.01867	0.01776	0.0174
	CFEF (mg/g)	4.7	6.1	5.6	6.9

**Table 5:** Isotherm parameters for the sorption of 4-CP on the prepared 2:1 AC.

Model	Parameter	Temperature (K)			
		298	303	308	313
Langmuir	*q_mon_* (mg/g)	535.4	555.5	569.8	585.4
	*K_L_* (L/mg)	0.089	0.0875	0.0860	0.0844
	MPSD	6.6	7.2	5.4	3.3
Freundlich	*K* (l/mg)^1/n^(mg/g)	28.4	28.2	28.8	29.4
	*n*	1.03	1.01	1.02	1.02
	MPSD	24.6	28.6	35.6	35.9

**Table 6:** Comparison of the Langmuir monolayer adsorption capacity, qmon, of 4-CP on different adsorbents.

Adsorbent	qmon, mg/g	Reference
Refuse-derived fuel waste	540	[48]
Activated carbons from sewage sludge:	192.6	[22]
Activation *T* = 723 K,		
KOH:C = 1:1		
Activated carbons from sewage sludge:	231.4	[22]
Activation *T* = 1023 K,		
KOH:C = 1:1		
HDTMAB-montmorillonite	43.3	[49]
DDTMA-montmorillonite	5.57	[50]
DTAB-Mt	331.1	[51]
CTAB-Mt	395.0	[51]
PSN2	307.2	[21]
PSN2.5	401.1	[21]
PSN3	408.8	[21]
PSN3.5	424.2	[21]
AC from date pits 2:1 AC at	535.4	This work

**Table 7:** Thermodynamic parameters for adsorption of 4-CP on the prepared 2:1 AC.

∆*H*^0^ (J/mol)	∆*S*^0^ (J/mol·K)	*E_a_* (J/mol)	–∆*G*^0^ (J/mol)			
			298 K	303 K	308 K	313 K
1474.2	25.1	261.7	5868.1	6008.5	6250.8	6499.9

## Abbreviations

The following abbreviations are used in this manuscript:

4-CP	4-Chlorophenol
AC	Activated Carbon
ASAP	Accelerated Surface Area and Porosimetry
BET	Brunauer–Emmett–Teller theory
CFEF	Composite Fractional Error Function
MPSD	Marquarrdt’s Percent Standard Deviation
SEM	Scanning Electron Microscope
